# Supplementary figures and images for: Non-cell-autonomous regulation of germline proteostasis by insulin/IGF-1 signaling-induced dietary peptide uptake via PEPT-1
Source: EMBO J. 2024 Sep 16;43(21):7. doi: 10.1038/s44318-024-00234-x (PMC11535032; doi:10.1038/s44318-024-00234-x)

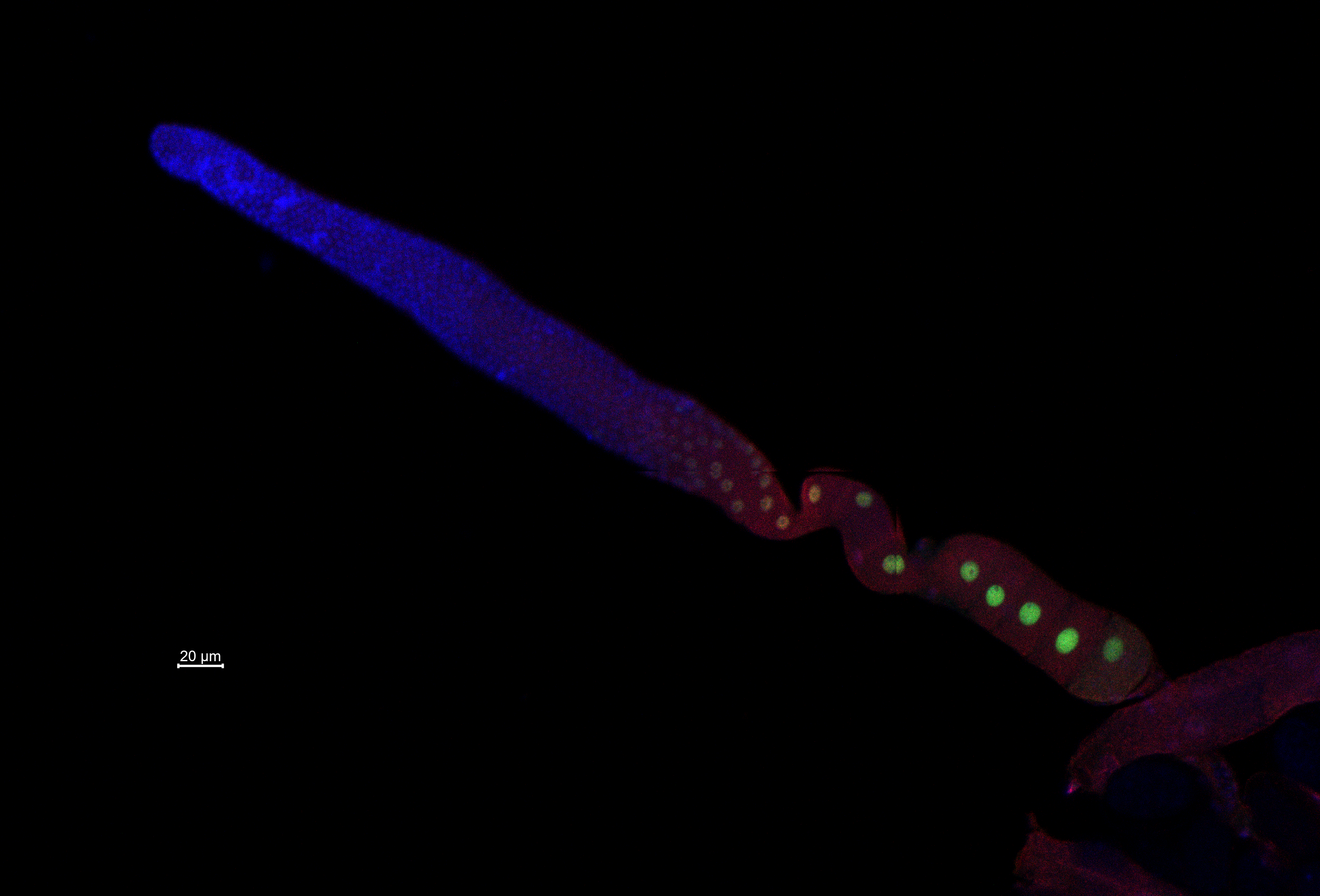

Supplement: Supplementary file 2 — Source data Fig. 1 [file 44318_2024_234_MOESM2_ESM.zip › Figure 1/1F/control_red-FK2_green-H3.tif]

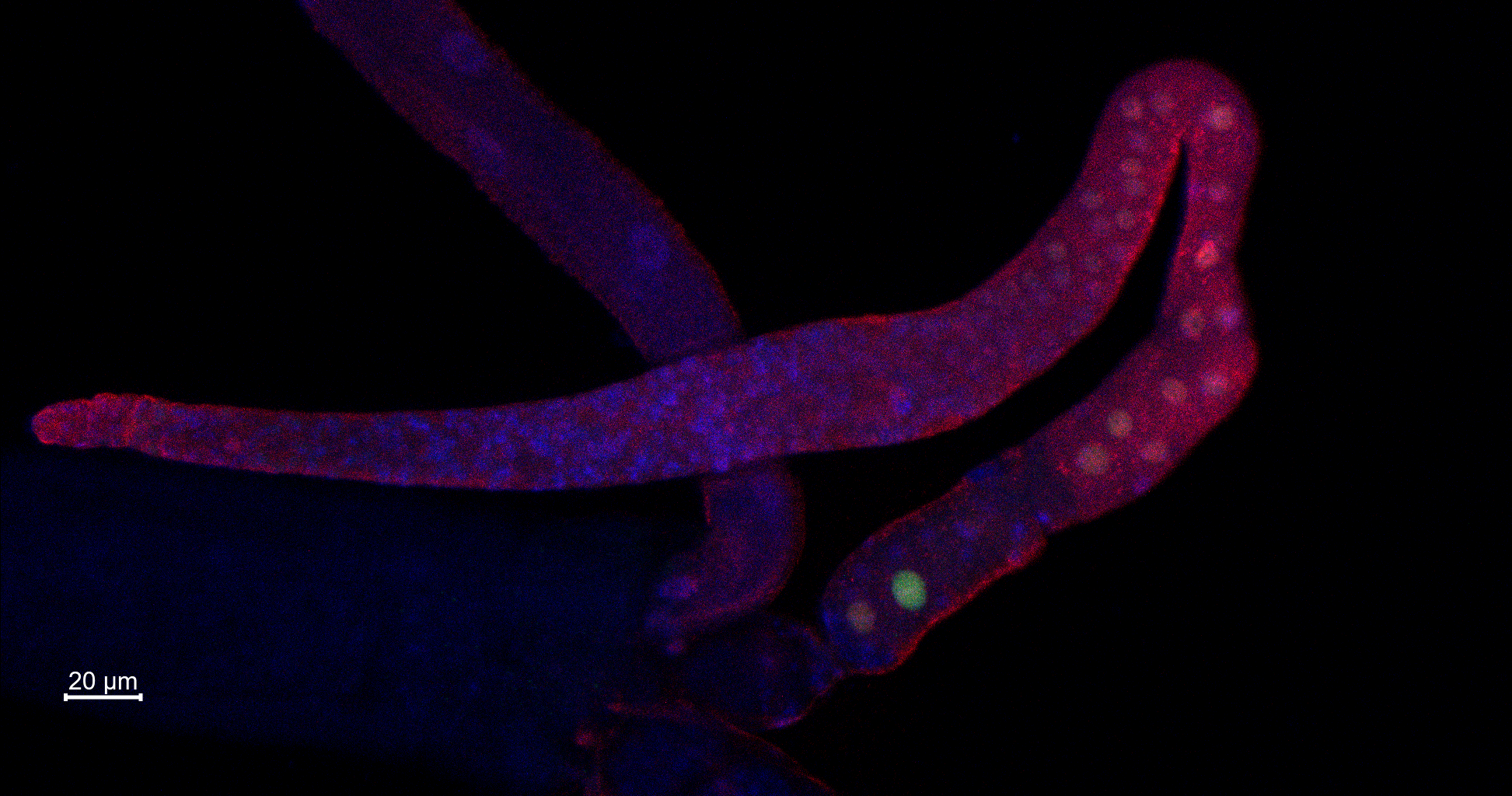

Supplement: Supplementary file 2 — Source data Fig. 1 [file 44318_2024_234_MOESM2_ESM.zip › Figure 1/1F/HSF-1 depl_red-FK2_green-H3.tif]

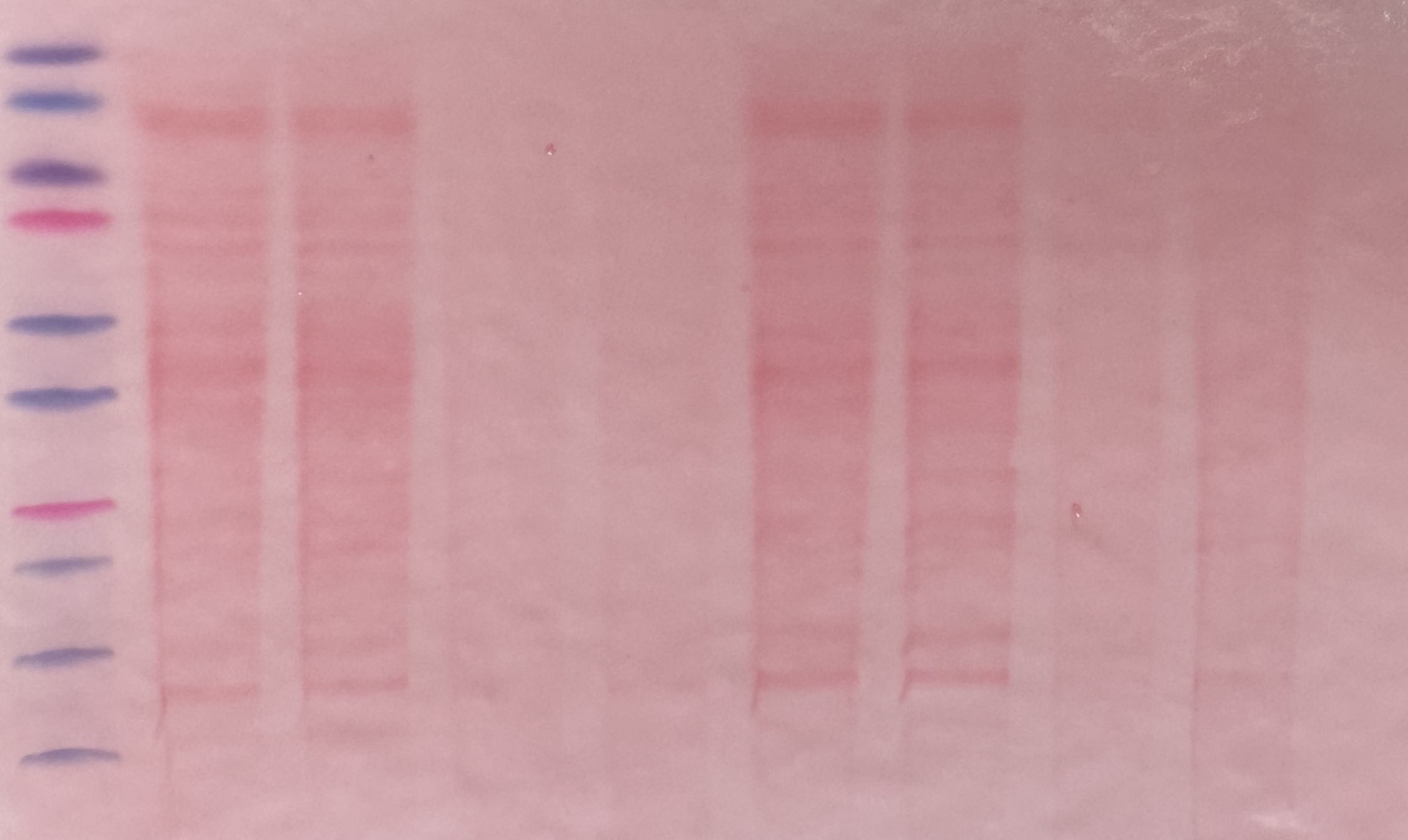

Supplement: Supplementary file 2 — Source data Fig. 1 [file 44318_2024_234_MOESM2_ESM.zip › Figure 1/1I/Ponceau.jpg]

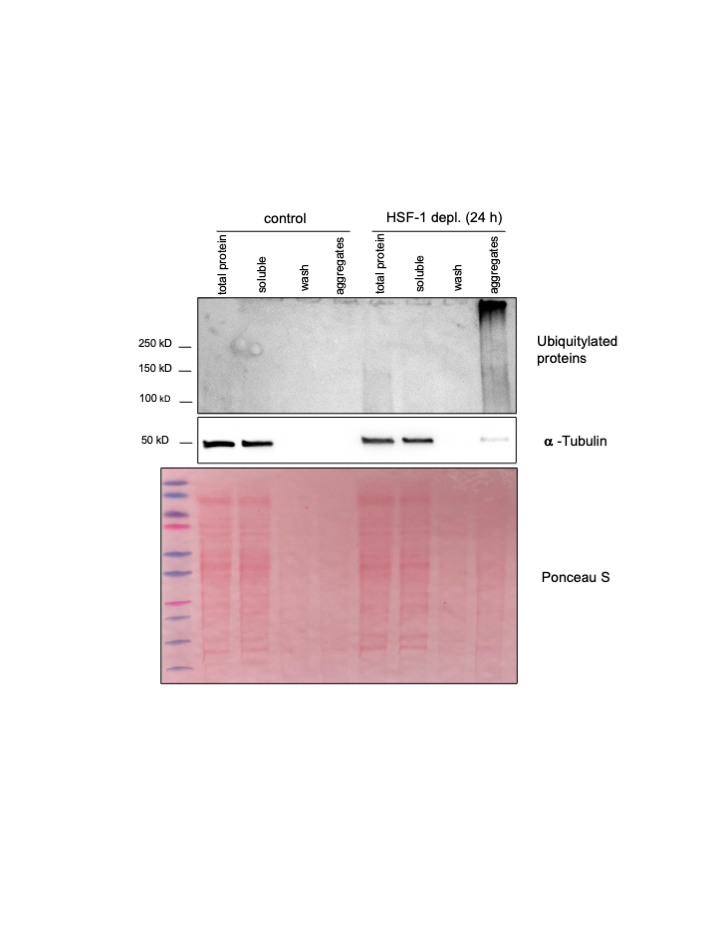

Supplement: Supplementary file 2 — Source data Fig. 1 [file 44318_2024_234_MOESM2_ESM.zip › Figure 1/1I/western in 1I with labeling.tiff]

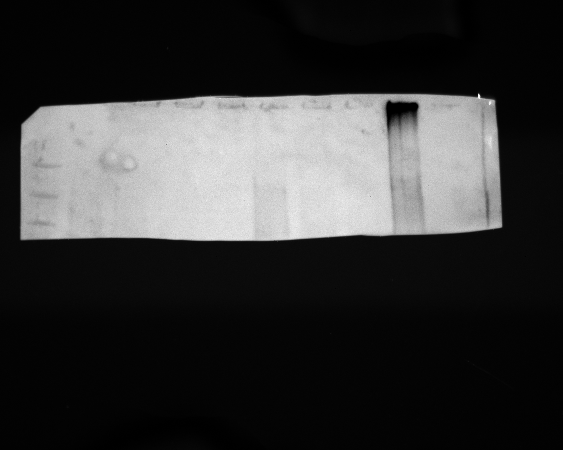

Supplement: Supplementary file 2 — Source data Fig. 1 [file 44318_2024_234_MOESM2_ESM.zip › Figure 1/1I/Ubiquitin_above 75kd.tif]

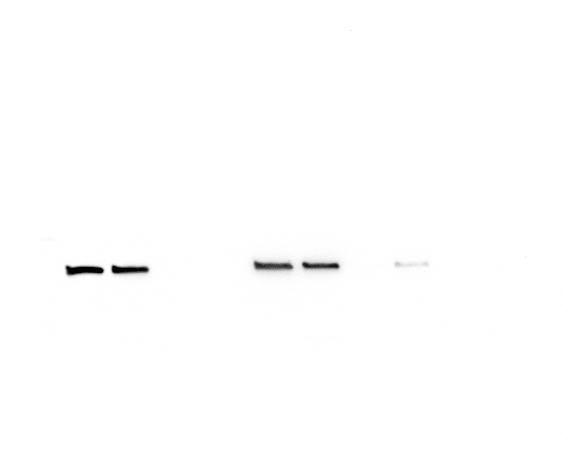

Supplement: Supplementary file 2 — Source data Fig. 1 [file 44318_2024_234_MOESM2_ESM.zip › Figure 1/1I/TUBULIN_below 75kd.tif]

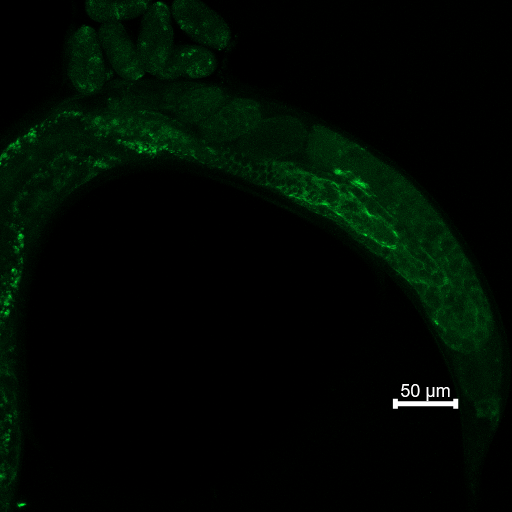

Supplement: Supplementary file 2 — Source data Fig. 1 [file 44318_2024_234_MOESM2_ESM.zip › Figure 1/1C/nmy-2_HSF-1 depl_PI.tif]

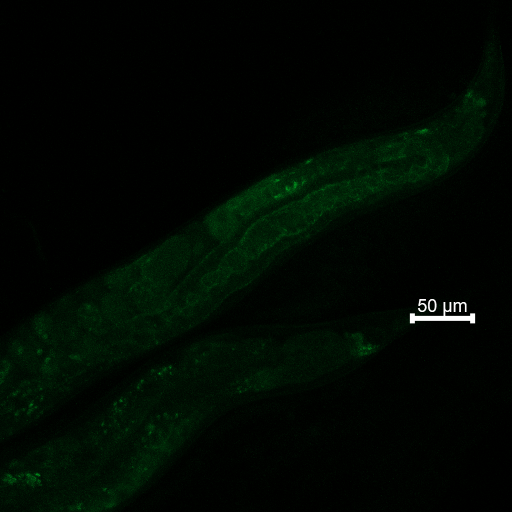

Supplement: Supplementary file 2 — Source data Fig. 1 [file 44318_2024_234_MOESM2_ESM.zip › Figure 1/1C/nmy-2_HSF-1 depl_no PI.tif]

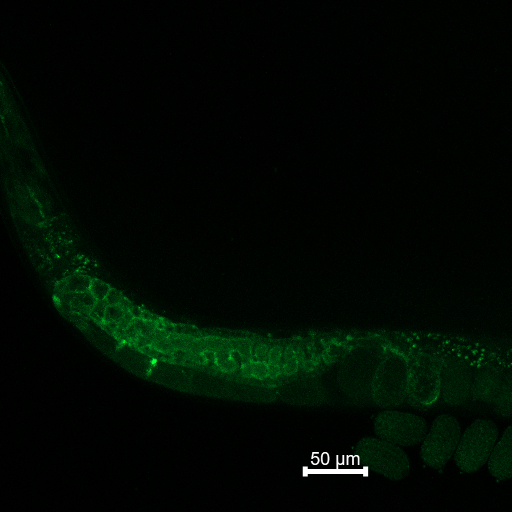

Supplement: Supplementary file 2 — Source data Fig. 1 [file 44318_2024_234_MOESM2_ESM.zip › Figure 1/1C/nmy-2_control_PI.tif]

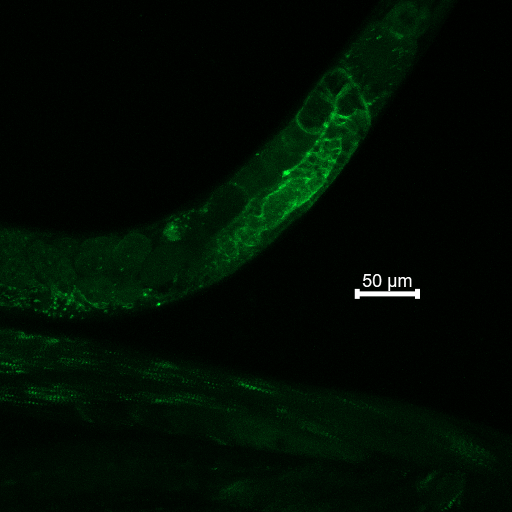

Supplement: Supplementary file 2 — Source data Fig. 1 [file 44318_2024_234_MOESM2_ESM.zip › Figure 1/1C/nmy-2_control_no PI.tif]

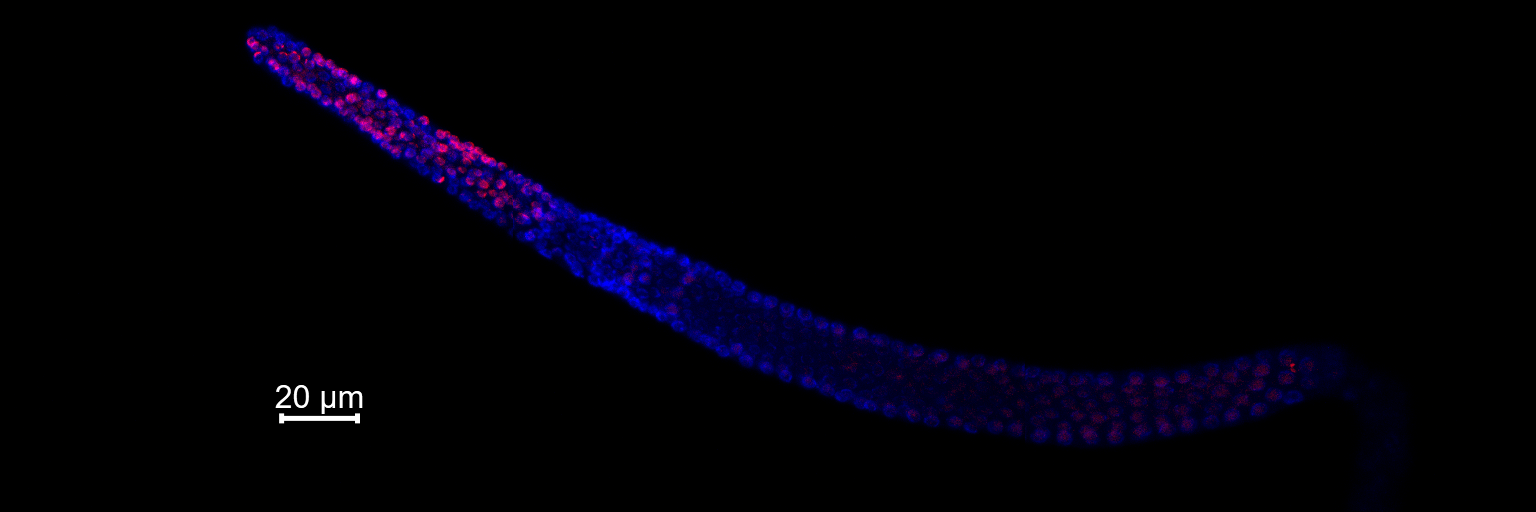

Supplement: Supplementary file 3 — Source data Fig. 2 [file 44318_2024_234_MOESM3_ESM.zip › Figure 2/2A/HSF-1 depl_16h.tif]

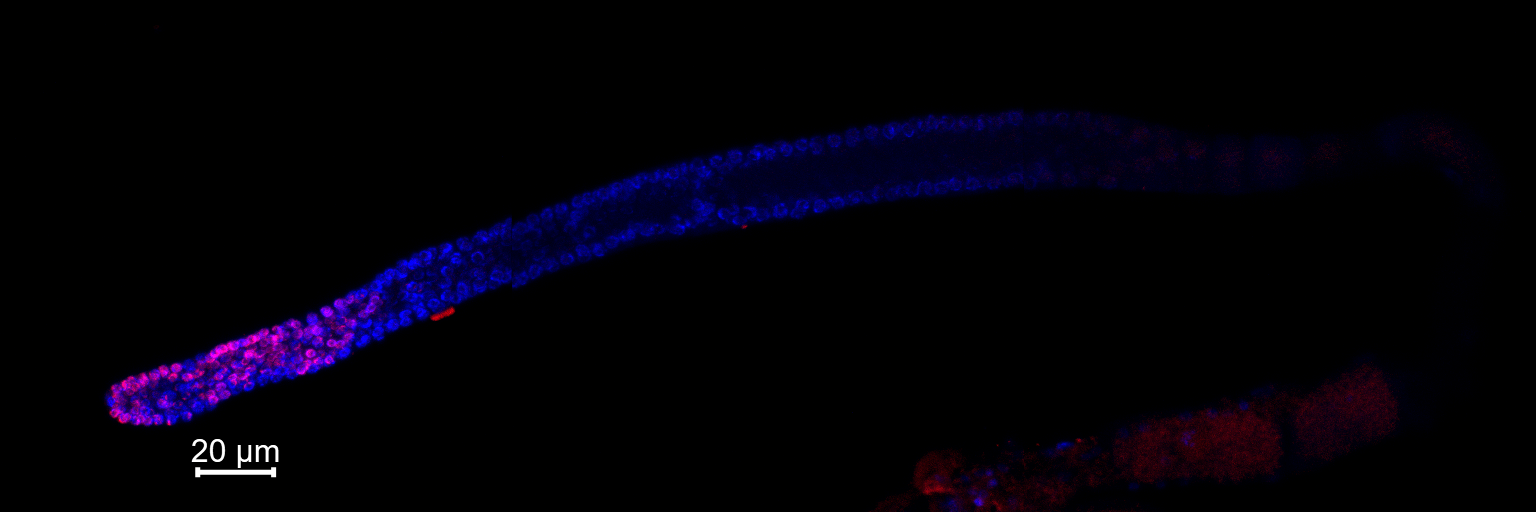

Supplement: Supplementary file 3 — Source data Fig. 2 [file 44318_2024_234_MOESM3_ESM.zip › Figure 2/2A/control_16h.tif]

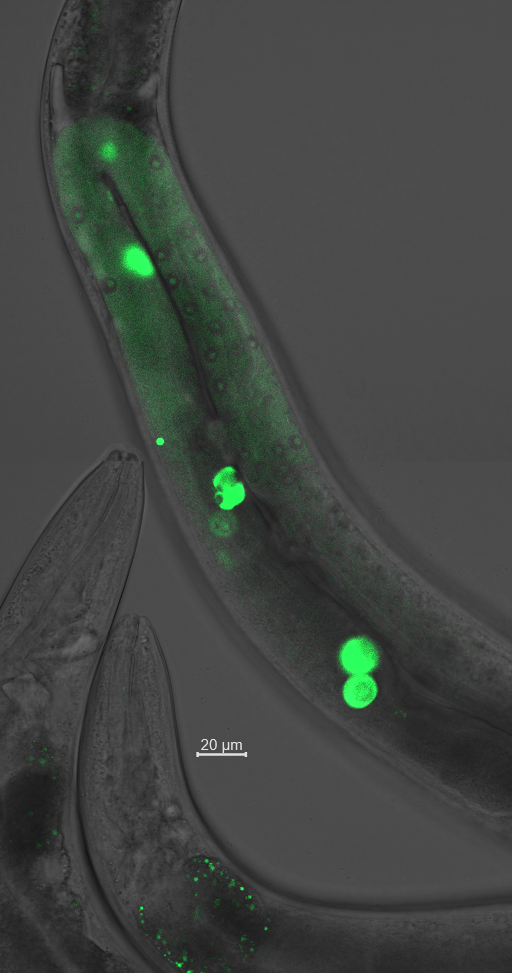

Supplement: Supplementary file 3 — Source data Fig. 2 [file 44318_2024_234_MOESM3_ESM.zip › Figure 2/2C/HSF-1 depl_AO staining.tif]

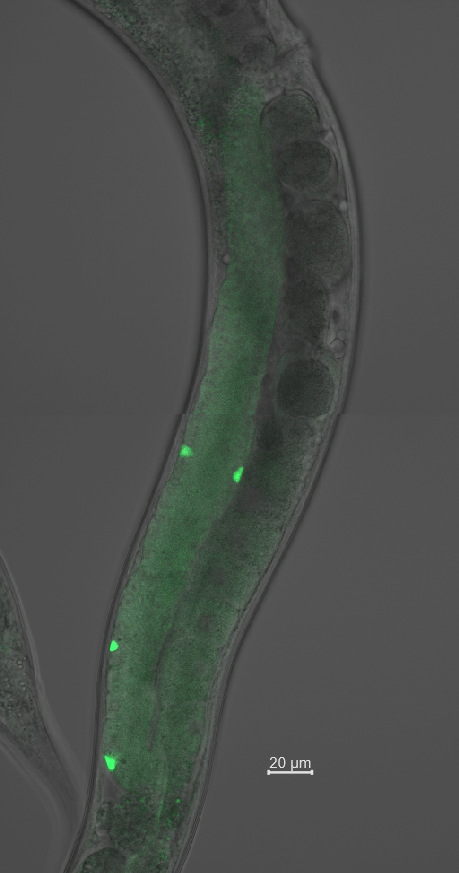

Supplement: Supplementary file 3 — Source data Fig. 2 [file 44318_2024_234_MOESM3_ESM.zip › Figure 2/2C/control_AO staining.tif]

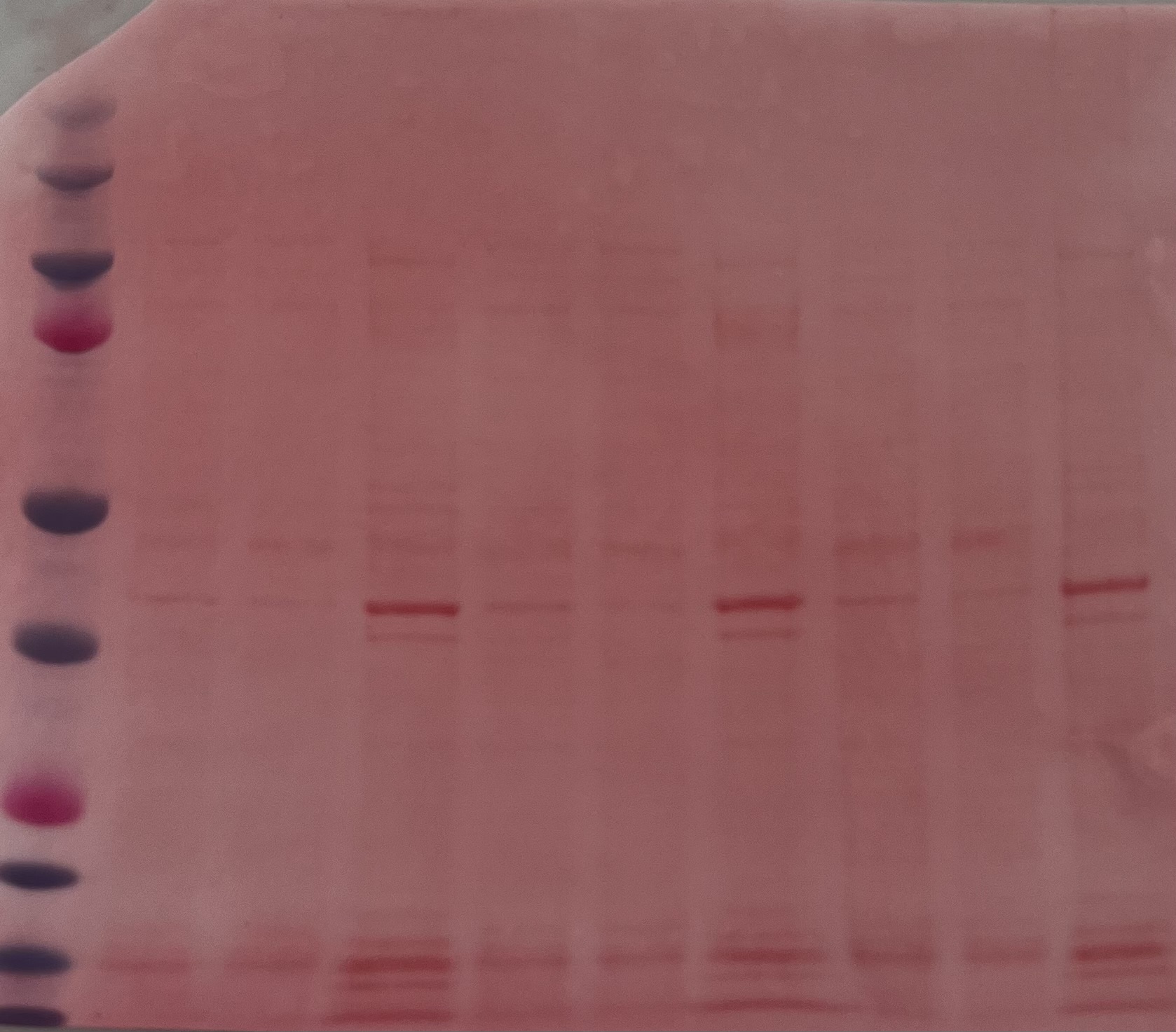

Supplement: Supplementary file 4 — Source data Fig. 3 [file 44318_2024_234_MOESM4_ESM.zip › Figure 3/3H/ponceau_daf2.jpg]

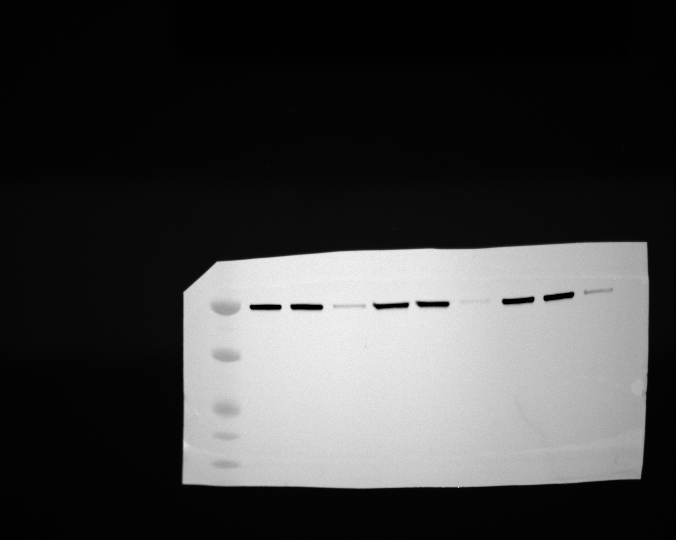

Supplement: Supplementary file 4 — Source data Fig. 3 [file 44318_2024_234_MOESM4_ESM.zip › Figure 3/3H/tubulin_below 75kd_daf2.tif]

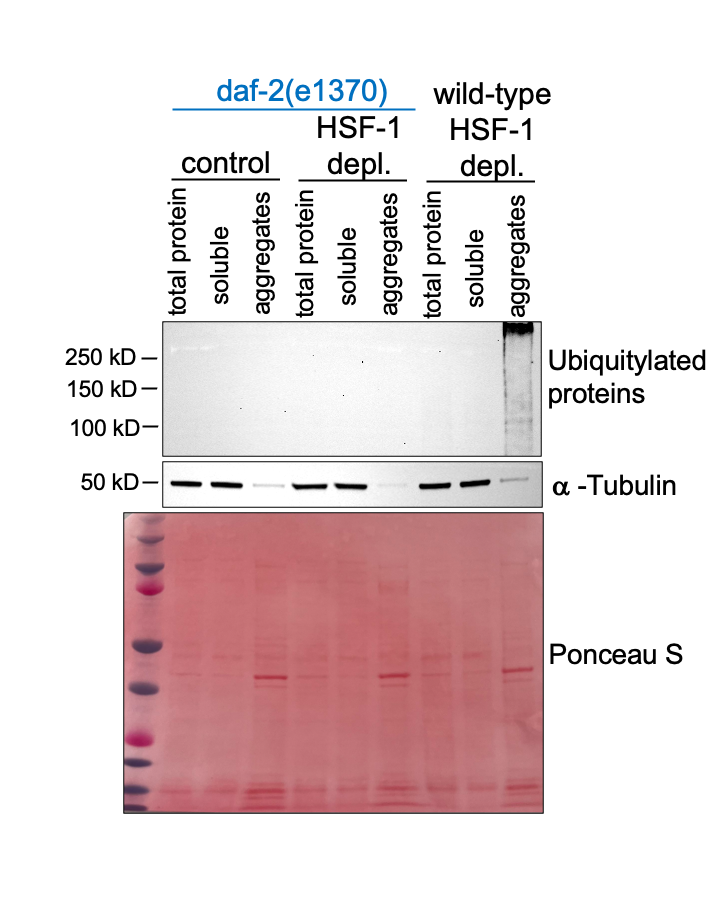

Supplement: Supplementary file 4 — Source data Fig. 3 [file 44318_2024_234_MOESM4_ESM.zip › Figure 3/3H/3H_daf-2 aggregation western with labeling.tiff]

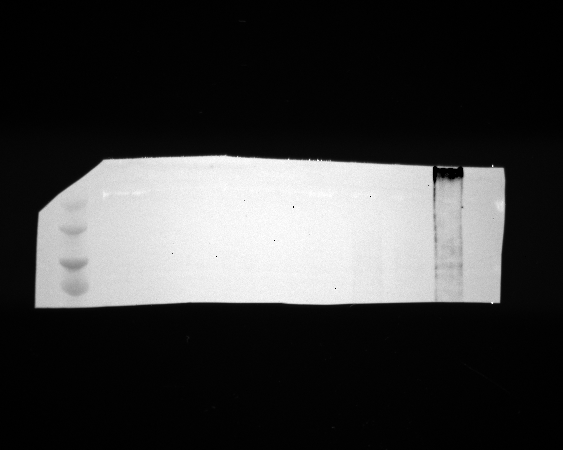

Supplement: Supplementary file 4 — Source data Fig. 3 [file 44318_2024_234_MOESM4_ESM.zip › Figure 3/3H/ubiqutin_75kD and above_daf2.tif]

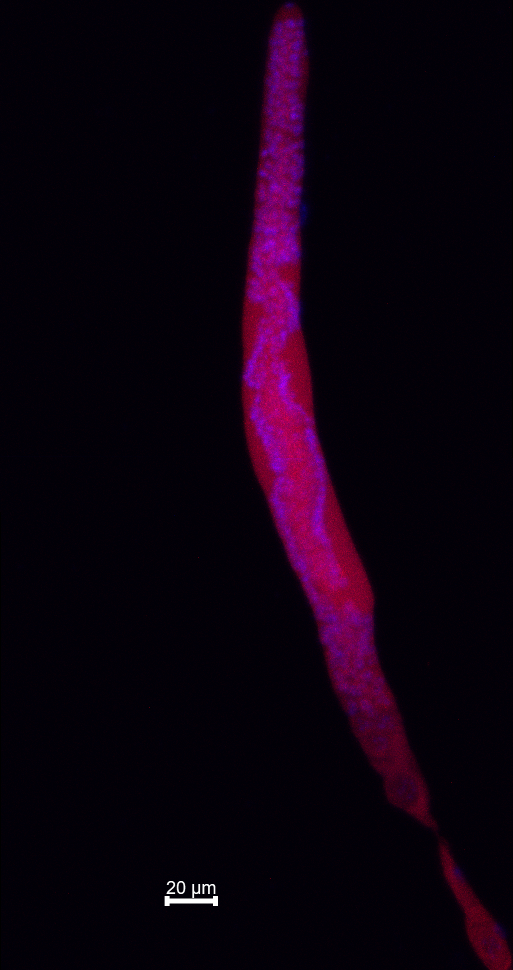

Supplement: Supplementary file 5 — Source data Fig. 4 [file 44318_2024_234_MOESM5_ESM.zip › Figure 4/4E/wild-type(N2_L4440).tif]

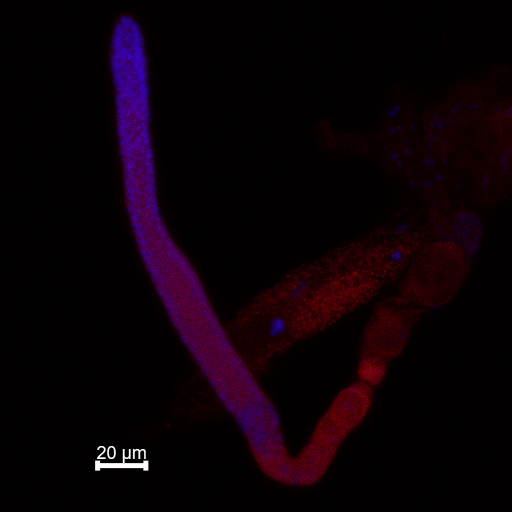

Supplement: Supplementary file 5 — Source data Fig. 4 [file 44318_2024_234_MOESM5_ESM.zip › Figure 4/4E/daf-2(e1370)_control RNAi(L4440).tif]

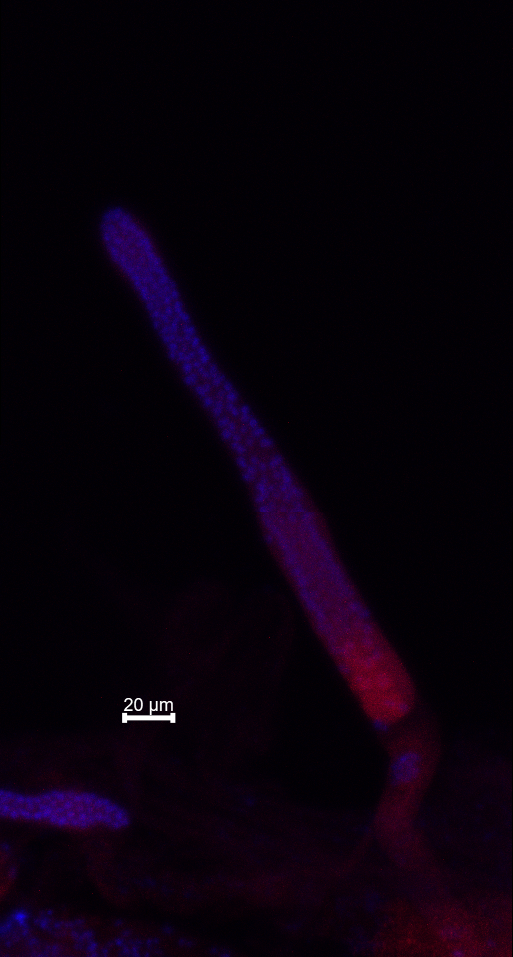

Supplement: Supplementary file 5 — Source data Fig. 4 [file 44318_2024_234_MOESM5_ESM.zip › Figure 4/4E/daf-2(e1370)_daf-2 RNAi.tif]

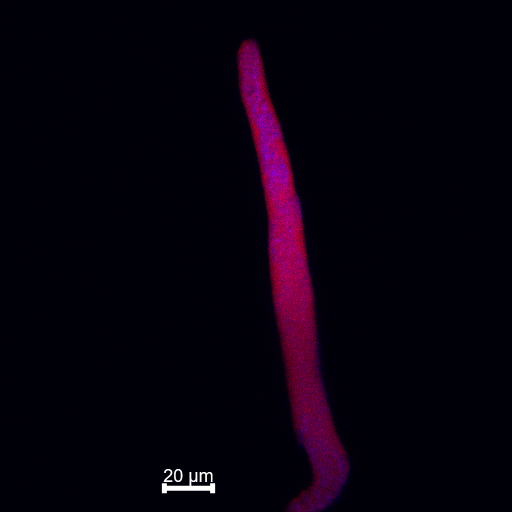

Supplement: Supplementary file 5 — Source data Fig. 4 [file 44318_2024_234_MOESM5_ESM.zip › Figure 4/4E/daf-2(e1370)_daf-16 RNAi.tif]

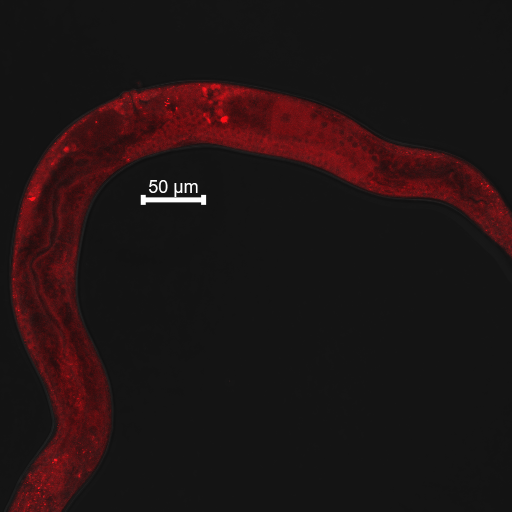

Supplement: Supplementary file 5 — Source data Fig. 4 [file 44318_2024_234_MOESM5_ESM.zip › Figure 4/4C/rps6_daf2.tif]

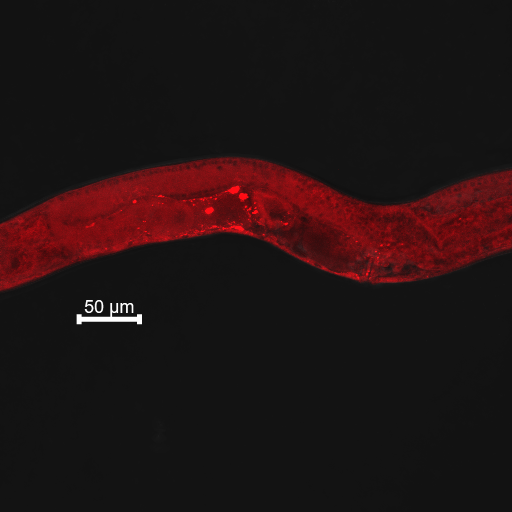

Supplement: Supplementary file 5 — Source data Fig. 4 [file 44318_2024_234_MOESM5_ESM.zip › Figure 4/4C/rps6_wild-type.tif]

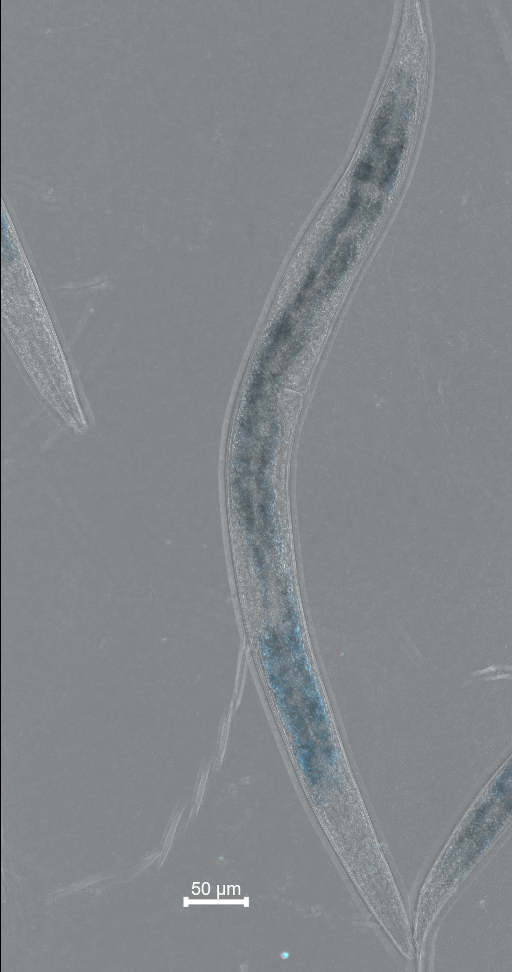

Supplement: Supplementary file 8 — Source data Fig. 7 [file 44318_2024_234_MOESM8_ESM.zip › Figure 7/7C/daf-2(e1370)_daf-2 RNAi_AMCA.tif]

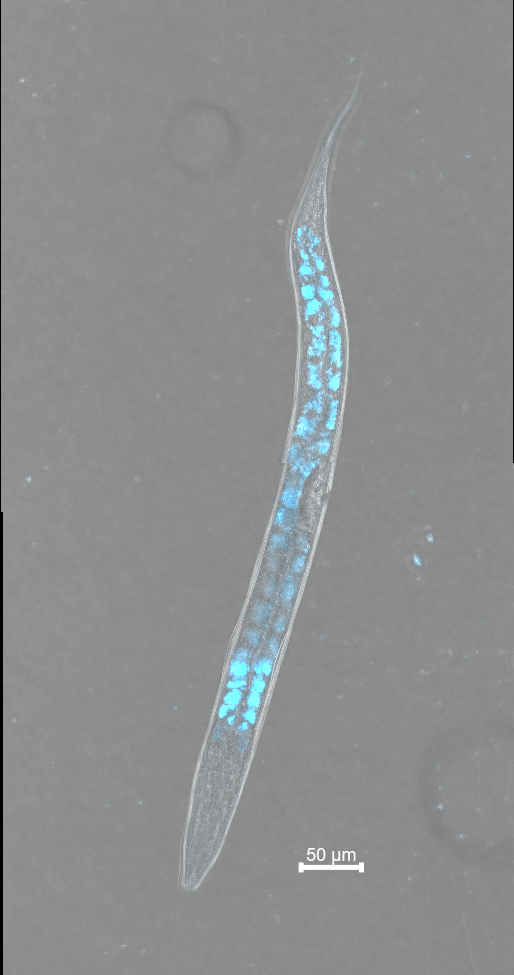

Supplement: Supplementary file 8 — Source data Fig. 7 [file 44318_2024_234_MOESM8_ESM.zip › Figure 7/7C/wild-type_control RNAi (L4440)_AMCA.tif]

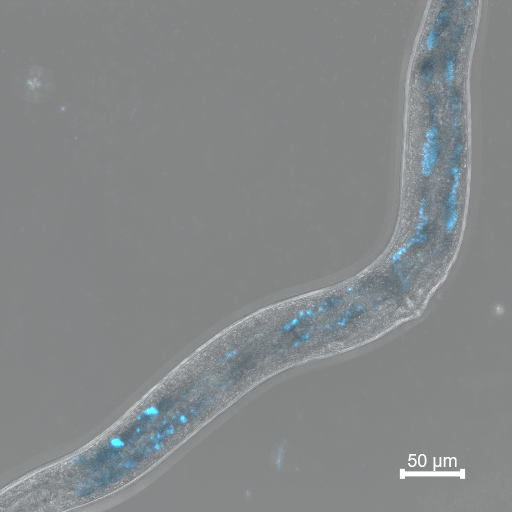

Supplement: Supplementary file 8 — Source data Fig. 7 [file 44318_2024_234_MOESM8_ESM.zip › Figure 7/7C/daf-2(e1370)_control RNAi(L4440)_AMCA.tif]

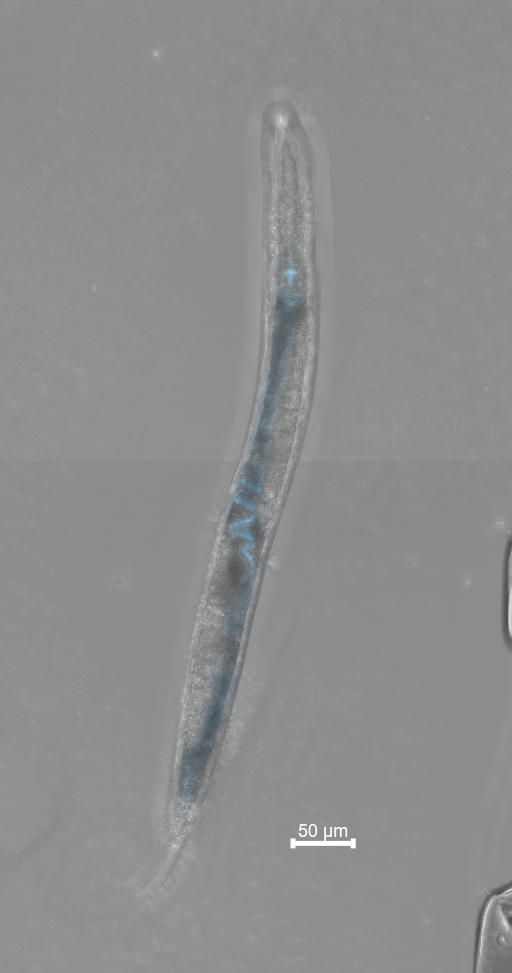

Supplement: Supplementary file 8 — Source data Fig. 7 [file 44318_2024_234_MOESM8_ESM.zip › Figure 7/7A/Pept-1 RNAi_AMCA.tif]

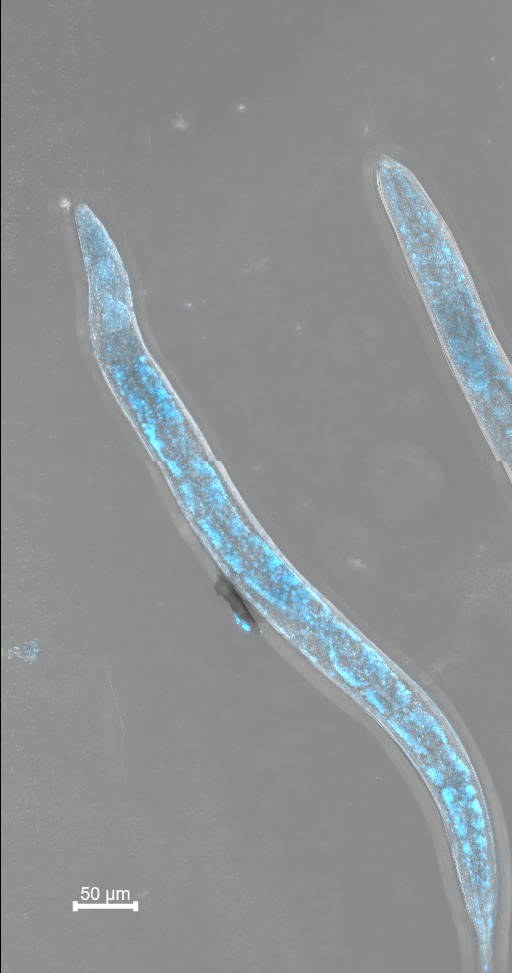

Supplement: Supplementary file 8 — Source data Fig. 7 [file 44318_2024_234_MOESM8_ESM.zip › Figure 7/7A/control RNAi_AMCA.tif]
