# Supplementary material for: Non-cell-autonomous regulation of germline proteostasis by insulin/IGF-1 signaling-induced dietary peptide uptake via PEPT-1
Source: EMBO J. 2024 Sep 16;43(21):7. doi: 10.1038/s44318-024-00234-x (PMC11535032; doi:10.1038/s44318-024-00234-x)
Supplement: Supplementary file 9 — Expanded View Figures [file 44318_2024_234_MOESM9_ESM.pdf]

Expanded View Figures

Figure EV1. Related to Fig. 1. Loss of HSF-1 from the germline impairs the expression of chaperone genes, causing protein degradation and aggregation.

(**A**) Histograms showing mRNA fold changes (FC) of the *nmy-2* gene upon HSF-1 depletion from the germline of young adults for 8 h, 16 h, or 24 h based on RNA-seq analysis. The chaperone gene *hsp-90* and proteasome component *pas-1* are included as controls. Mean and standard deviation are plotted (8 h & 16 h: $N = 4$; 24 h: $N = 3$). (**B**) Representative images of GFP::H2B transgene in the germline upon HSF-1 depletion. HSF-1 was depleted from the germline of young adults using AID for 24 h in the presence or absence of the proteasome inhibitor bortezomib (PI) for the last 6 h of HSF-1 depletion. The dashed lines outline the gonads. The white asterisks (*) indicate the distal end of gonads where progenitor cells are located. The arrows indicate the fully grown oocytes, where the levels of GFP::H2B are quantified. (**C**) Representative images of the endogenously tagged HSF-1::degron::GFP upon auxin-induced degradation. HSF-1 was depleted from the germline of young adults upon auxin treatment for 24 h in the presence or absence of the proteasome inhibitor bortezomib (PI) for the last 6 h of HSF-1 depletion. The dashed lines outline the gonads. The white asterisks (*) indicate the distal end of gonads where progenitor cells are located. The proteasome inhibitor treatment was not sufficient to restore HSF-1 protein levels. (**D–F**) Representative images of NMY-2::GFP transgene in the germline upon HSF-1 depletion from germ cells starting at the young-adult stage for 24 h (**D**) and 48 h (**E**), and the corresponding quantification (**F**). The dashed lines outline the gonads. The white asterisks (*) indicate the distal end of gonads where progenitor cells are located. In the dot plots, the mean and standard deviation are plotted (Day 2: $N = 8$; Day 3: control $N = 7$, HSF-1 depletion $N = 9$). *P*-values were calculated by unpaired t-test. ns: $p >= 0.05$.

 © The Author(s)

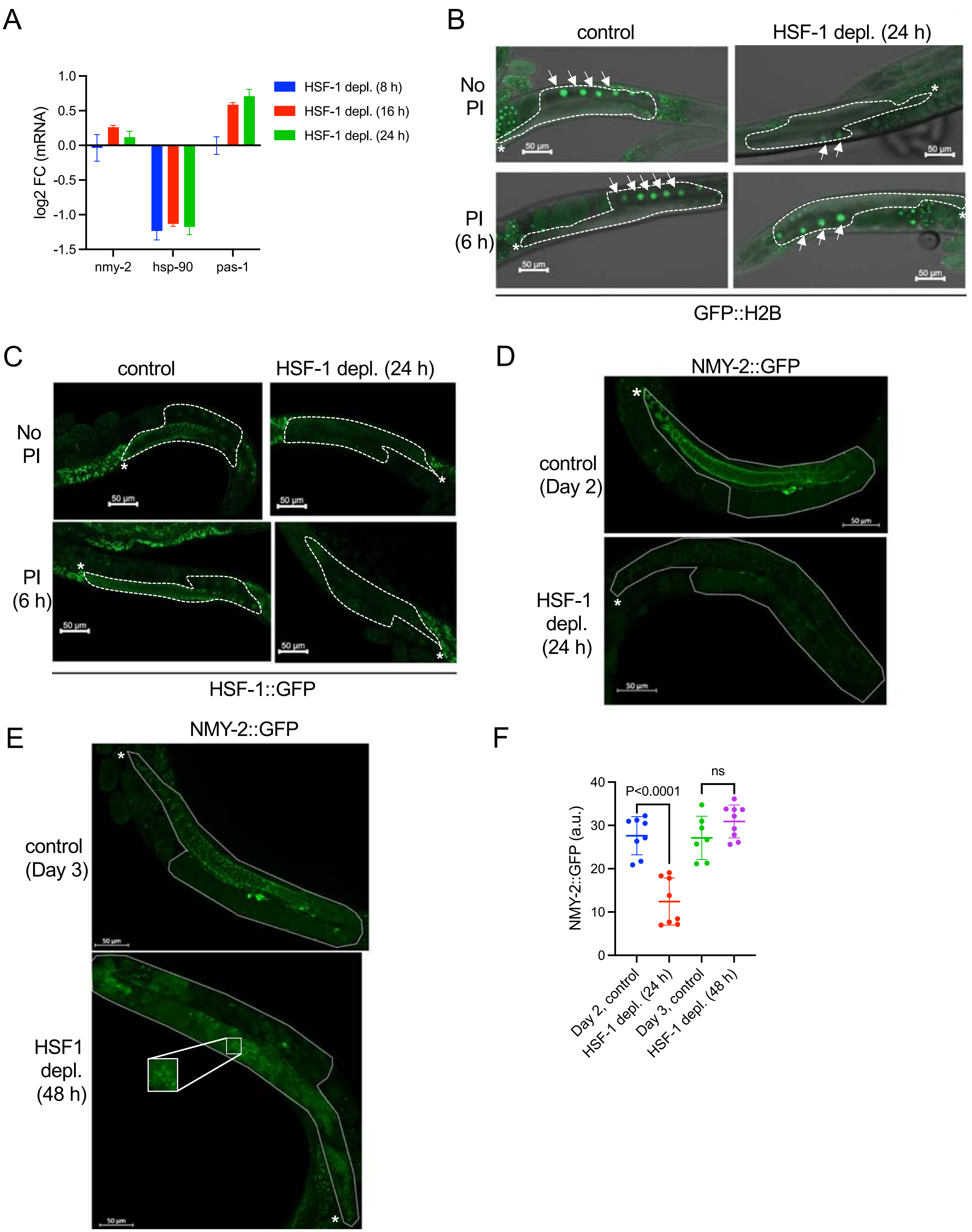
A
1.0
0.5
0.0
-0.5
-1.0
-1.5
log2 FC (mRNA)
nmy-2
hsp-90
pas-1
HSF-1 depl. (8 h)
HSF-1 depl. (16 h)
HSF-1 depl. (24 h)
B
control
HSF-1 depl. (24 h)
No PI
PI (6 h)
50 μm
GFP::H2B
C
control
HSF-1 depl. (24 h)
No PI
PI (6 h)
50 μm
HSF-1::GFP
D
NMY-2::GFP
control (Day 2)
HSF-1 depl. (24 h)
50 μm
E
NMY-2::GFP
control (Day 3)
HSF1 depl. (48 h)
50 μm
F
NMY-2::GFP (a.u.)
40
30
20
10
0
P<0.0001
ns
Day 2, control
HSF-1 depl. (24 h)
Day 3, control
HSF-1 depl. (48 h)

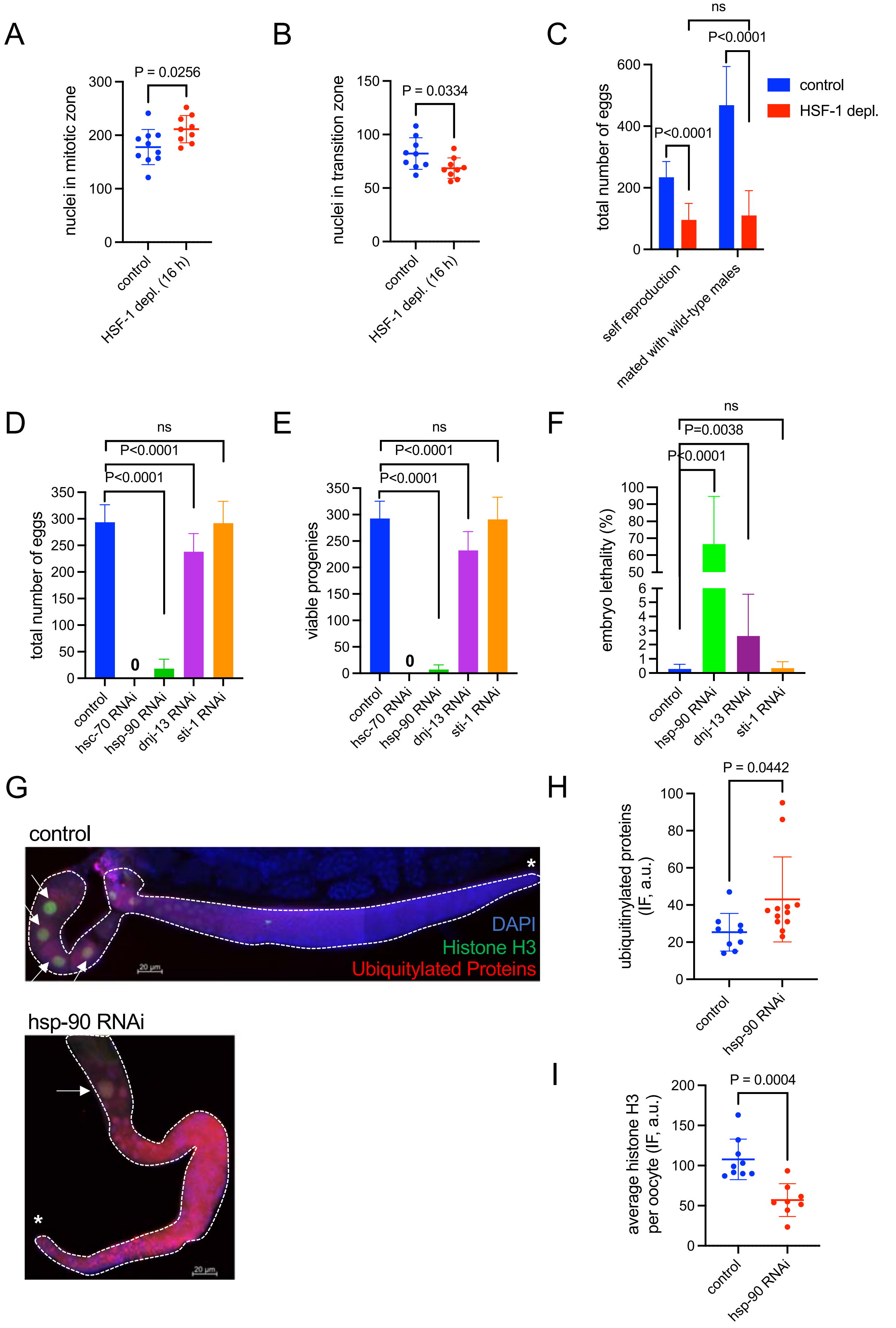

A
P = 0.0256
nuclei in mitotic zone
control
HSF-1 depl. (16 h)
B
P = 0.0334
nuclei in transition zone
control
HSF-1 depl. (16 h)
C
ns
P<0.0001
P<0.0001
total number of eggs
control
HSF-1 depl.
self reproduction
mated with wild-type males
D
ns
P<0.0001
P<0.0001
total number of eggs
0
control
hsc-70 RNAi
hsp-90 RNAi
dnj-13 RNAi
sti-1 RNAi
E
ns
P<0.0001
P<0.0001
viable progenies
0
control
hsc-70 RNAi
hsp-90 RNAi
dnj-13 RNAi
sti-1 RNAi
F
ns
P=0.0038
P<0.0001
embryo lethality (%)
control
hsp-90 RNAi
dnj-13 RNAi
sti-1 RNAi
G
control
DAPI
Histone H3
Ubiquitylated Proteins
hsp-90 RNAi
H
P = 0.0442
ubiquitinylated proteins (IF, a.u.)
control
hsp-90 RNAi
I
P = 0.0004
average histone H3 per oocyte (IF, a.u.)
control
hsp-90 RNAi

 © The Author(s)

Figure EV2. Related to Fig. 2. HSF-1 is required in the adult germline for fecundity and oocyte quality.

(**A**, **B**) Quantification of the total number of nuclei in the mitotic zone (**A**) and transition zone (**B**) upon depletion of HSF-1 from the germline of young adults for 16 h. Mean and standard deviation are plotted ($N >= 9$). *P*-values were calculated by unpaired t-test. (**C**) Histograms showing the total number of eggs (live and dead) upon HSF-1 depletion from the germline of hermaphrodites starting from Day 1 of adulthood during self-reproduction and when mated with N2 males. Mean and standard deviation are plotted (self-reproduction: $N = 15$; mating experiments: $N >= 10$). *P*-values were calculated by unpaired t-test. ns: $p >= 0.05$. (**D–F**) Histograms showing the total number of eggs (live and dead) (**D**), viable progenies (**E**), and embryo lethality (**F**) with germline-specific RNAi against selective chaperone and co-chaperone genes. The four selected chaperone and co-chaperone genes are direct target genes of HSF-1, which have HSF-1 binding at their promoters in germ cells (ChIP-seq) and significantly decrease mRNA expression upon 8 h of HSF-1 depletion from the germline (FDR: 0.05, RNA-seq) (Edwards et al, 2021). Animals were treated with control RNAi (L4440) or RNAi against chaperone and co-chaperone genes starting from egg lay. Mean and standard deviation are plotted ($N >= 15$). *P*-values were calculated by unpaired t-test. ns: $p >= 0.05$. (**G–I**) Representative images (**G**) and quantification of ubiquitylated proteins (not free ubiquitin) (**H**) and endogenous histone H3 (**I**) by immunofluorescence (IF) upon germline-specific RNAi against *hsp-90*. Animals were treated with control RNAi (L4440) or RNAi against *hsp-90* starting from the L3 larval stage, which allowed more oocytes to develop in the hsp-90 RNAi group than starting the RNAi treatment at egg lay. IF was performed on Day 1 of adults. The dashed lines outline the gonads with the white asterisks (*) marking the distal end. The arrows indicate the fully grown oocytes, where the levels of histone H3 are quantified. Mean and standard deviation are plotted (control RNAi: $N = 9$; hsp-90 RNAi: $N = 12$ for ubiquitylated proteins and $N = 8$ for histone H3 as not all hsp-90 RNAi treated animals developed fully grown oocytes). *P*-values were calculated by unpaired t-test.

© The Author(s)

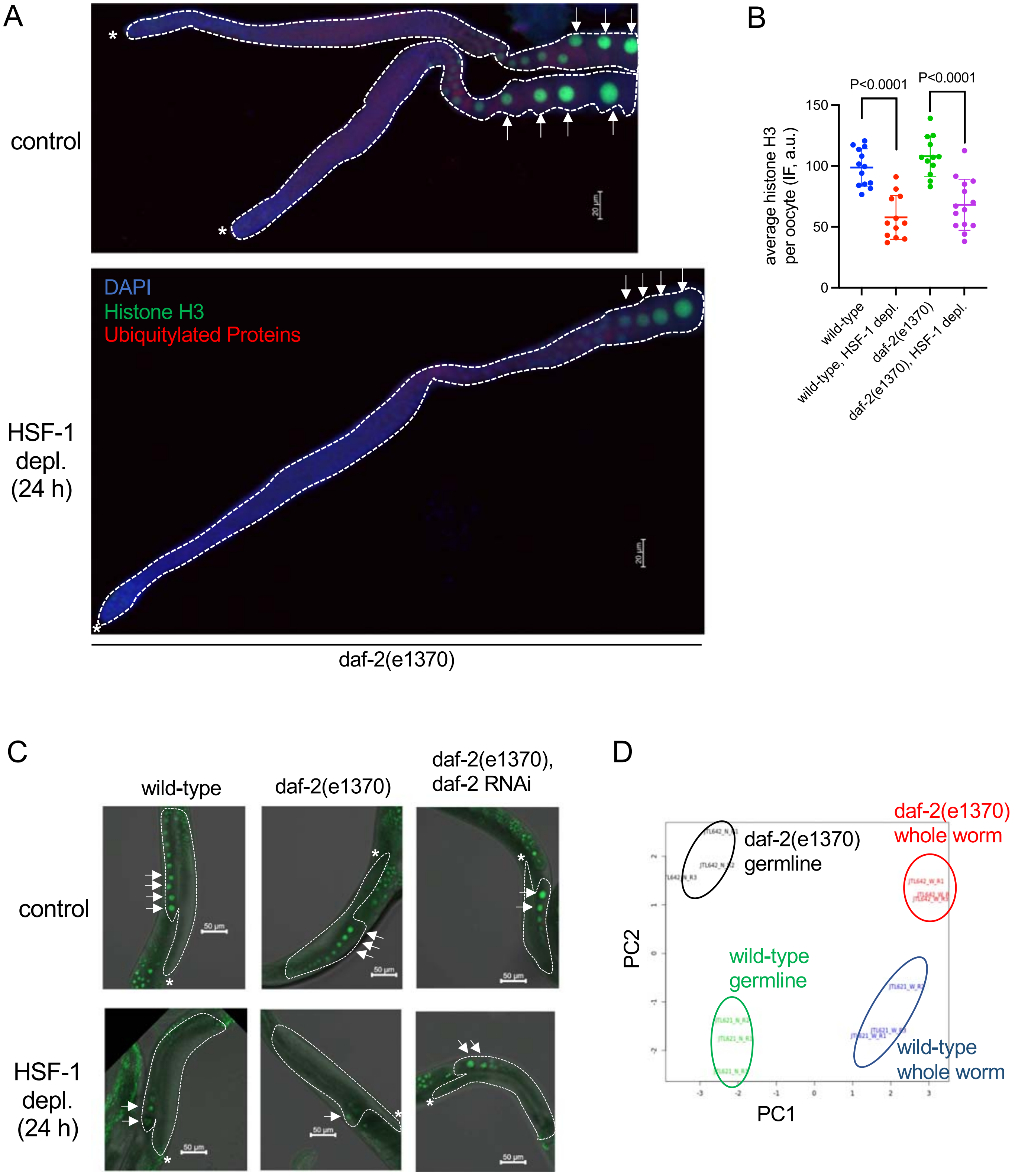
A
control
HSF-1
depl.
(24 h)
DAPI
Histone H3
Ubiquitylated Proteins
daf-2(e1370)
B
P<0.0001
P<0.0001
average histone H3
per oocyte (IF, a.u.)
150
100
50
0
wild-type
wild-type, HSF-1 depl.
daf-2(e1370)
daf-2(e1370), HSF-1 depl.
C
wild-type
daf-2(e1370)
daf-2(e1370),
daf-2 RNAi
control
HSF-1
depl.
(24 h)
D
daf-2(e1370)
germline
daf-2(e1370)
whole worm
wild-type
germline
wild-type
whole worm
PC2
PC1

 © The Author(s)

Figure EV3. Related to Fig. 3 and Fig. 4. Reduced Insulin/IGF-1 signaling (IIS) confers resilience against limited protein folding capacity in gametogenesis through the regulation of translation.

(**A**, **B**) Representative images (**A**) of endogenous histone H3 and ubiquitylated proteins (not free ubiquitin) by immunofluorescence (IF) upon germline-specific depletion of HSF-1 in the *daf-2(e1370)* animals starting from the young-adult stage for 24 h. The dashed lines outline the gonads with the white asterisks (*) marking the distal end. The levels of histone H3 in the fully grown oocytes (as indicated by arrows) are quantified (**B**) together with the results from the wild-type animals as a control. Mean and standard deviation are plotted ($N >= 12$). *P*-values were calculated by unpaired t-test. (**C**) Representative images of GFP::H2B transgene in fully grown oocytes upon germline-specific depletion of HSF-1 for 24 h from young adults. The wild-type and *daf-2(e1370)* animals were treated with *daf-2* RNAi or control RNAi (L4440) starting from egg lay. The dashed lines outline the gonads with the white asterisks (*) marking the distal end. The arrows indicate the fully grown oocytes, where the levels of GFP::H2B are quantified. (**D**) Principal component analysis (PCA) on the RNA-seq results of the whole worm lysate or isolated germline nuclei from young adults of the wild-type and *daf-2(e1370)* animals ($N = 3$).

© The Author(s)

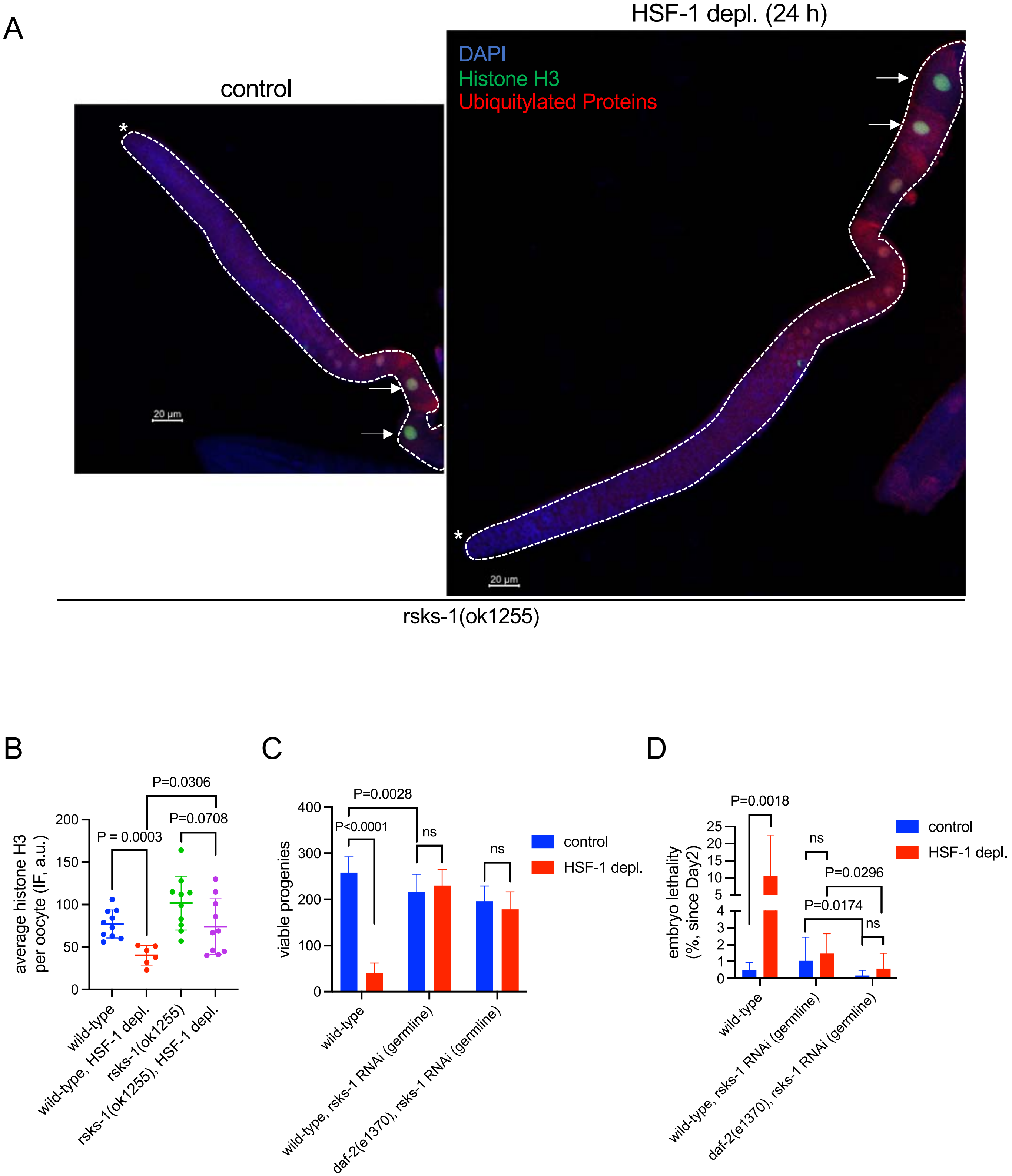
A
control
HSF-1 depl. (24 h)
DAPI
Histone H3
Ubiquitylated Proteins
20 µm
20 µm
rsks-1(ok1255)
B
average histone H3 per oocyte (IF, a.u.)
P = 0.0003
P=0.0306
P=0.0708
wild-type
wild-type, HSF-1 depl.
rsks-1(ok1255)
rsks-1(ok1255), HSF-1 depl.
C
viable progenies
P=0.0028
P<0.0001
ns
ns
control
HSF-1 depl.
wild-type
wild-type, rsks-1 RNAi (germline)
daf-2(e1370), rsks-1 RNAi (germline)
D
embryo lethality (%, since Day2)
P=0.0018
ns
P=0.0296
P=0.0174
ns
control
HSF-1 depl.
wild-type
wild-type, rsks-1 RNAi (germline)
daf-2(e1370), rsks-1 RNAi (germline)

 © The Author(s)

Figure EV4. Related to Fig. 5. Reduced translation rate underlies robust germline proteostasis and resilience against proteotoxic stress mediated by low insulin/IGF-1 signaling (IIS).

(**A**, **B**) Representative images (**A**) of endogenous histone H3 and ubiquitylated proteins (not free ubiquitin) by immunofluorescence (IF) upon germline-specific depletion of HSF-1 in the *rsks-1(ok1255)* animals starting from the young-adult stage for 24 h. The dashed lines outline the gonads with the white asterisks (*) marking the distal end. The levels of histone H3 in the fully grown oocytes (as indicated by arrows) are quantified (**B**) together with the results from the wild-type animals as a control. The mean and standard deviation are plotted (wild-type, HSF-1 depletion: $N = 6$; the other three groups: $N = 10$). *P*-values were calculated by unpaired t-test. (**C**, **D**) Histograms showing the brood size (**C**) and embryo lethality (**D**) of the wild-type and *daf-2(1370)* animals treated with germline-specific *rsks-1* RNAi and with depletion of HSF-1 from the germline. The treatment of control RNAi (L4440) or RNAi against *rsks-1* started at the egg lay. HSF-1 depletion from the germline was initiated on Day 1 of adulthood. Embryo lethality was measured on Day 2 and after. Mean and standard deviation are plotted ($N >= 15$). *P*-values were calculated by unpaired t-test. ns: $p >= 0.05$.

© The Author(s)

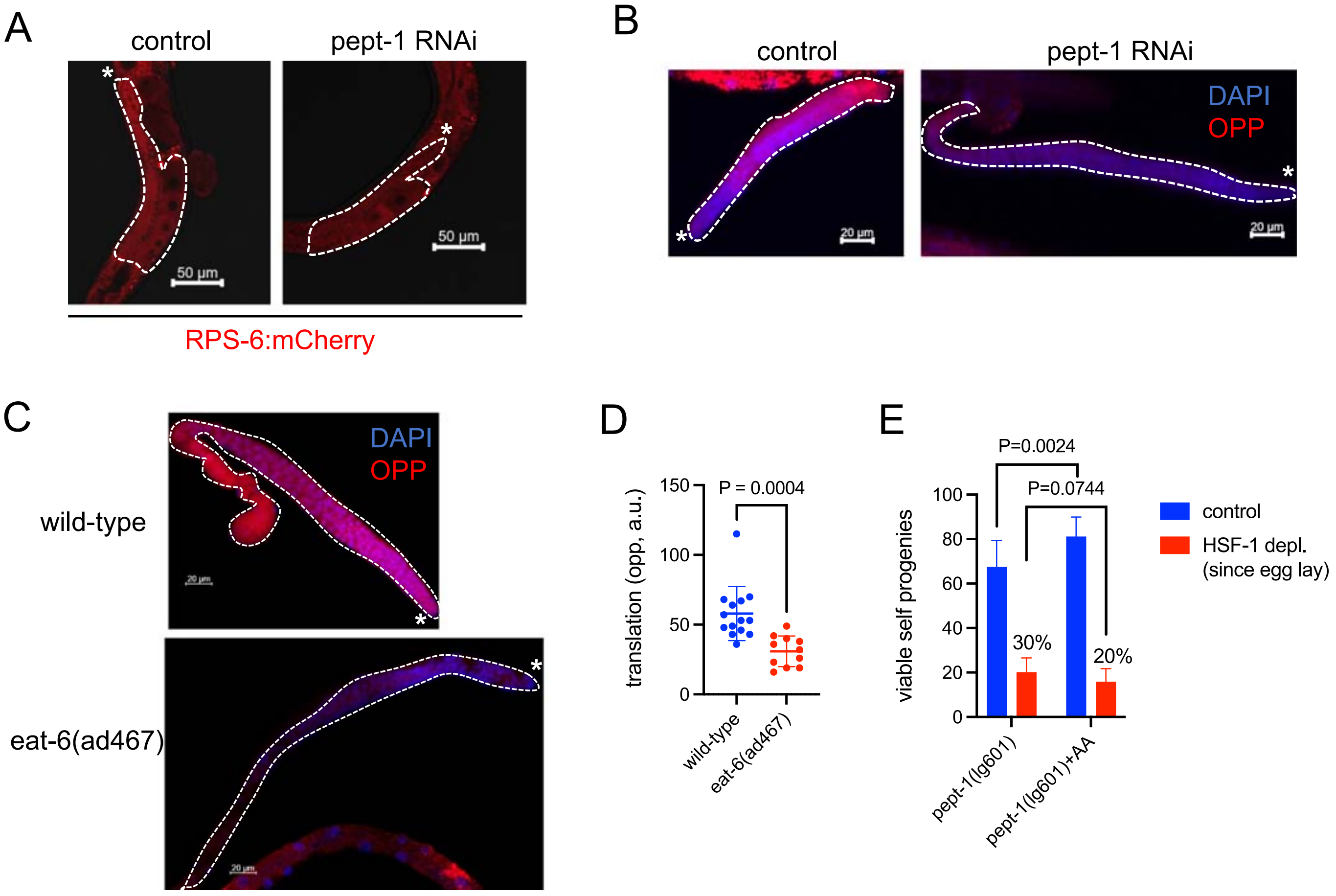

Figure EV5. Related to Fig. 7. Insulin/IGF-1 signaling (IIS) regulates germline protein synthesis and proteostasis via peptide uptake in the intestine.

(**A**, **B**) Representative images of the endogenously tagged RPS-6::mCherry protein (**A**) and translation measured by OPP incorporation (**B**) in the germline upon *pept-1* RNAi. RNAi started at egg lay, and measurement was done in young adults. The dashed lines outline the gonads with the white asterisks (*) marking the distal end. (**C**, **D**) Representative images (**C**) and quantification (**D**) of translation measured by OPP incorporation in the germline of the wild-type and *eat-6(ad467)* animals. The *eat-6(ad467)* mutant serves as a dietary restriction model. The translation assay was done in young adults. The dashed lines outline the gonads with the white asterisks (*) marking the distal end. Mean and standard deviation are plotted (wild-type: $N = 14$; *eat-6(ad467)*: $N = 11$). The *P*-value was calculated by unpaired t-test. (**E**) Brood size analysis of the *pept-1(lg601)* animals in the presence or absence of amino acid (AA) supplements. Both depletion of HSF-1 from the germline and the supplement of AA started from egg lay. Mean and standard deviation are plotted ($N >= 12$). *P*-values were calculated by unpaired t-test. The percentage of brood size obtained upon HSF-1 depletion compared with the corresponding control is labeled.

 © The Author(s)
